# Deep venous thrombosis and atypical antipsychotics: three cases report

**DOI:** 10.1186/2008-2231-20-71

**Published:** 2012-10-31

**Authors:** Fatemeh Sheikhmoonesi, Seyyedeh Fatemeh Bahari Saravi

**Affiliations:** 1 Psychiatry and Behavioral Sciences Research Center, Mazandaran University of Medical Sciences, Sari, Iran

**Keywords:** Atypical antipsychotics, Deep venous thrombosis, Psychiatry, Olanzapine, Risperidone

## Abstract

**Background:**

Deep venous Thrombosis is a serious, possible life threatening event which is often ignored in psychiatric Settings.

**Purpose:**

In this paper three cases of deep venous Thrombosis (DVT) following the use of olanzapine and risperidone are presented.

**Methods:**

The data of Three patients was collected from hospital records.

**Results:**

The patients were in good general physical health and had no personal or familial history of DVT. The patients were not overweight (BMI < 25) but they suffered from DVT after initiating risperidone and olanzapine.

**Conclusion:**

Risk of DVT exists in patients under treatment with atypical antipsychotics in spite of no pre existing risk factor.

## Introduction

Deep venous thrombosis (DVT) and pulmonary embolism (PE) are frequent illnesses with an annual incidence of 1 per 1000 persons and a mortality rate above 15% in the first 3 months after diagnosis [1]. Risk of DVT is relatively more in psychiatric patients rather than people who are mentally healthy [2], especially in Paients suffering from schizophrenia and bipolar disorder [3]. In a case reported by Zornberg & Jick, a significantly increased risk of DVT was seen in patients receiving first generation antipsychotics, particularly in the first months of the treatment [4]. It is believed that Antipsychotics may induce pathological blood clotting via sedating them as well as reducing motor activity, metabolic Syndrome such as obesity and hyperprolactinemia [5].

The patients’ sedation, immobilization or psychopathological symptoms, often interfere with the diagnosis of DVT, therefore, it may not always be recognized at first [6].

In this report three psychiatric patients who developed DVT after using olanzapine and risperidon are presented. No personal and familial history of DVT was found in these cases. Nevertheless, their condition suggests a possible association between atypical antipsychotics and DVT in the absence of any pre- existing risk factors. Written informed consents were obtained from the patients for publication of this report.

## Case 1

The patient was a 38- year- old female with a diagnosis of depressive episode in course of bipolar 1 disorder,according to DSM-IV-TR presenting with depressed mood, food refusal and isolation.she had discontinued her drugs since 4 weeks before.

Routine physical examination and laboratory tests were normal. There was not familial history of psychiatric disorder. The patient was not overweight (body mass index: 22). She also had no identified cardiovascular risk factor. Due to the intensity of symptoms she underwent ECT (electroconvulsive therapy) and received Risperidone 4 mg/daily and Biperiden 2 mg/daily. Two weeks later swelling was seen in her right leg. A diagnosis of DVT was suspected and she was initiated on high dose low-molecular-weight heparin. After 24 hours of initiation of the treatment, patient died due to cardiac arrest.

## Case 2

A 24- year- old female was admitted to hospital due to a psychotic disorder (NOS) according to DSM-IV-TR. She had visual and auditory hallucination and persecutory delusion. Three years prior to this admission, the patient was diagnosed with paranoid schizophrenia. However she did not have a good compliance. Therefore she had been admitted Several times in psychiatric ward. When she referred to our hospital, she have not received any drug for two months. She was treated with electroconvulsive therapy (ECT), Risperidone 6 mg/day and Biperiden 4 mg/daily. After two months she complained of severe pain in her right leg. Clinical examination revealed right lower limb swelling. Doppler ultrasonography and Contrast venography in the right lower limb confirmed the diagnosis of DVT, she underwent thrombectomy and consequently swelling was resolved.

## Case 3

A 64 – year – old male was referred to psychiatric ward due to aggression, grandiose delusion, hyposomnia (decreased need for sleep) and flight of idea . His medical history showed that he was diagnosed with bipolar 1 disorder according to DSM-IV-TR,around ten years prior to this study.on this last admission, he initiated on sodium valproate 1200 mg/day, olanzapine 15 mg/day, biperiden 4 mg/day. After three weeks, he suffered from swelling in left leg. All of the mentioned drugs were discontinued which led to his full recovery.

## Discussion

The link between conventional antipsychotic medications and venous Thromboembolism (VTE) was first suggested in the 1950s. However, there are few case reports maintaining an association between atypical antipsychotics and VTE and they are mainly related to Clozapine [7]. Atypical antipsychotics have been associated with sedation, a more sedentary lifestyle and weight gain, all of which are predisposing factors for VTE [8]. However, these are unlikely to be etiologic factors in early thromboembolic occurrence.

A previous case study described three elderly patients (an 89- year – old male, a 78 – year – old male and an 83- year – old female) in whom olanzapine therapy was associated with VTE [9]. But the cases presented in this paper are different since all three were under 65 years old. Therefore, it could be suggested that Olanzapine could result in thrombosis at any age.

In another case report massive pulmonary embolism was found in a 27- year – old obese Somali man, temporally related to ingestion of olanzapine over dose (nearly 150 mg) [10].

Thromboembolic complications have not previously been reported in patients taking low doses of olanzapine.

So far, the biological mechanisms responsible for this possible adverse drug reaction have not been found, but a number of hypotheses have been made . For instance, antipsychotics like olanzapine and risperidone that are antagonist of 5 HT_2_ receptors, so, they can induce increase of serotonin which in turn might provoke enhanced aggregation of platelets, thereby increasing the risk for thrombosis [11].

Patients being treated with olanzapine and risperidone should be monitored clinically for venous thromboembolism to ensure early detection and intervention, and a possible discontinuation of treatment with olanzapine and risperidone should be considered if the diagnosis of venous thromboembolism is made.

## Competing interests

The author(s) declare that they have no competing interest.

## Authors’ contributions

FS and SFBS have been involved in the acquisition of clinical data and in the reviewing the scientific literature.FS wrote the manuscript. Both authors read and approved the final manuscript.
